# *Sapium ellipticum* (Hochst.) Pax Ethanol Leaf Extract Maintains Lipid Homeostasis in Streptozotocin-Induced Diabetic Rats

**DOI:** 10.1155/2017/6463139

**Published:** 2017-06-07

**Authors:** Osasenaga Mcdonald Ighodaro, Oluseyi Adeboye Akinloye, Regina Ngozi Ugbaja, Samuel Olatunbosun Omotainse

**Affiliations:** ^1^Department of Biochemistry, Faculty of Sciences, Lead City University, Ibadan, Nigeria; ^2^Department of Biochemistry, College of Biosciences, Federal University of Agriculture, Abeokuta (FUNAAB), Abeokuta, Nigeria; ^3^College of Veterinary Medicine, Federal University of Agriculture, Abeokuta (FUNAAB), Abeokuta, Nigeria

## Abstract

Dyslipidemia is a common metabolic disorder especially in diabetes mellitus (DM). In this study, the ability of* Sapium ellipticum* (SE) leaf extract to restore lipid homeostasis in streptozotocin-induced diabetes was examined. DM was induced in experimental rats (Wistar strains) using single intraperitoneal dose (55 mg/kg body weight {BW}) of streptozotocin (STZ). Treatment of diabetic rats with SE was oral (p.o), at doses of 400 and 800 mg kg^−1^ BW, twice daily at 8 h interval for 21 days. Lipid parameters were analyzed in the serum of rats using test kits. SE caused a significant (*P* ≤ 0.05) reduction in STZ-induced hypercholesterolemia in a dose dependent pattern (13.7 and 17.89%). These effects were comparable to that provided by metformin (15.45%), a standard antidiabetic drug. Similar pattern was noted with serum triglycerides (TG) (10.63 and 19.06%) and LDL (31.47 and 25.97%). Adipose tissue TG level was improved to near normal. Besides, the cardiovascular risk predictors in terms of atherogenic index of plasma (AIP) and LDL/HDL ratio were lowered by 57.85 and 44.12%, respectively. However, the extract failed to significantly reverse the STZ-induced decline in serum HDL. Overall, with AIP value of 0.28 and LDL/HDL ratio of 0.91, SE demonstrated the potential to maintain lipid homeostasis in the diabetics.

## 1. Introduction

Hyperlipidemia is a common condition associated with a number of diseases such as diabetes, high blood pressure, and cardiovascular disorders, as either a risk, symptomatic, or complication factor. It is characterized by high plasma level of lipid molecules, basically, total cholesterol (T-chol), total triglyceride (TG), low density lipoprotein cholesterol (LDL-chol), and decreased level of high density lipoprotein cholesterol (HDL-chol) [1, 2]. In diabetic state, sequel to insulin deficiency or defectiveness, glucose availability in body cells is compromised. The attendant effect is massive flux of free fatty acids (FFA) into the liver, leading to accumulation of excess fatty acids in the hepatocytes. The fatty acids are then converted to triglycerides and cholesterol and released into circulation, resulting in increased levels of plasma triglycerides and cholesterol.

In addition, hepatic VLDL-chol production is also increased following free fatty acid accumulation in the liver. The Increased VLDL-chol and triglycerides cause a concomitant decrease in the level of HDL-chol and increase in the concentration of LDL-chol through activation of lipoprotein lipase and lecithin acyl-cholesterol transferase [3, 4].

Hyperlipidemia worsens diabetic condition by making the patient susceptible to associated complications like retinopathy and erectile dysfunction and diseases such as hypertension and stroke. Lipid homeostasis is therefore critical, not only to recovery from diabetes but to also prevent comorbidity particularly in terms of hypertension, which often leads to stroke and ultimately to sudden mortality.

Some commonly consumed herbs have been reported to promote reduction in blood lipids [5–8].* Sapium ellipticum* (Hochst.) Pax enjoy huge therapeutic application in the local treatment of a number of disease conditions [9, 10], including diabetes (ethnobotanical survey). It belongs to the family Euphorbiaceae and is commonly referred to as jumping seed tree.* S. ellipticum* is widely distributed in eastern and tropical Africa. In southwest part of Nigeria, particularly among the Ilorin indigenes, the plant is popularly known as* aloko-ạgbọ*.

A few scientific investigations have been carried out on it. Adesegun et al. [11] in their in vitro study credited substantial antioxidant properties to the stem bark extract of the plant. Cytotoxicity screening of selected Nigerian plants used in traditional cancer treatment on HT29 (colon cancer) and MCF-7 (breast cancer) cell lines (HeLa cervix adenocarcinoma cells) indicated that* Sapium ellipticum* leaf extract expressed the highest cytotoxic activity among other plants with anticancer potential which was comparable to the reference drug, cisplatin [12]. The phythochemical constituents, in vitro antioxidant capacities, and antiplasmodial activities of* Sapium ellipticum* stem bark extracts were documented by Nana et al. [13]. Edimealem and colleagues [14] in their study demonstrated the presence of lupeol, lupeol acetate, and stigmasterol in the stem bark extract of* Sapium ellipticum*. This present study sought to investigate the ability of the plant leaf extract to maintain lipid homeostasis in diabetes mellitus.

## 2. Materials and Methods

### 2.1. Collection of* Sapium ellipticum* Leaves

Fresh* Sapium ellipticum* (SE) leaves were harvested in the month of December, 2012, from a forest in a suburb of Ibadan, southwest of Nigeria. The harvested leaves were taxonomically authenticated by a botanist (Mr. T. K. Odewo) at the Lagos University Herbarium (LUH), Nigeria, where a specimen was deposited and assigned a voucher number, LUH 5423.

### 2.2. Preparation of* Sapium ellipticum* Leaf Extracts

The plant material was freed of extraneous materials; air dried at room temperature; and milled to a fine powder, using a Waring blender. 300 grams of the powdered sample was macerated in 2.5 liters of the extracting solvent (ethanol) at room temperature. The mixture was allowed to stand for 72 h and stirred intermittently with a glass rod to facilitate extraction. Sieving of the mixture was achieved with a muslin cloth (maximum pore size 2 mm). The resulting filtrate on sieving was further filtered through Whatman filter paper (number 42) and subsequently reduced in volume with a rotary evaporator at 40°C. Final elimination of solvent and drying was done using a regulated water bath at 40°C.

### 2.3. Induction of Diabetes Mellitus with Streptozotocin in Experimental Rats

Single intraperitoneal (i.p) dose (55 mg Kg^−1^ BW) of freshly prepared streptozotocin (STZ) was administered to a batch of normoglycaemic rats starved for 16 h. Diabetes (fasting blood sugar level ≥ 200 mg/dL) was confirmed in the animals, 72 h after streptozotocin injection [15] using Acutecheck active glucometer with disposable test strips.

### 2.4. Experimental Design and Management of Animals

Eight normoglycemic animals constituted a control group (group 1). Thirty-two diabetic Wistar rats were randomly assigned to four groups (groups 2, 3, 4, and 5) containing eight animals each. Group 1 animals were administered olive oil (0.5 mL) and served as normal control. Group 2 animals were left untreated and served as STZ control animals. Groups 3 and 4 were, respectively, treated with 400 and 800 of SE kg^−1^ BW and the last group was treated with metformin (METF, 12 mg kg^−1^ BW), a reference antidiabetic drug. All treatments were done orally (p.o), twice daily at 8 h interval for a period of 21 days. All procedures for maintenance and sacrifice (care and use) of animals were carried out according to the criteria outlined by the National Academy of Science published by the National Institute of Health [16].

### 2.5. Estimation of Serum Lipid of Rats

Total cholesterol (T-chol) and total triglycerides (TG), low density lipoprotein (LDL) cholesterol (LDL-C), and high density lipoprotein (HDL) cholesterol (HDL-C) were determined using test kits (Linear chemicals).

### 2.6. Estimation of Triglyceride Content in the Adipose Tissue of Rats

Triglyceride quantification in the adipose tissues of rats was performed according to the procedure described by Buttler et al. [17].

### 2.7. Estimation of Cardiovascular Risk Level

Cardiovascular indicators such as Plasma Atherogenic Index and HDL-LDL ratio were calculated from the values of total triglyceride (TG), high density lipoprotein cholesterol (HDL), and low density lipoprotein cholesterol (LDL) obtained in Section 2.5 using the formulae

(1) Plasma  Atherogenic  Index=log10⁡Total  TGHDL-cholHDL-LDL  ratio=Plasma  HDL-cholPlasma  LDL-chol.

### 2.8. Qualitative Phytochemical Evaluation of SE Ethanol Leaf Extract

Standard procedures as described by Sofowora [18], Edeoga et al. [19], Trease and Evans [20], and Harborne [21] were used with some modifications to determine the phytochemicals present in SE extract.

### 2.9. Statistical Analysis of Data

Data analysis was performed using statistical software, Graphpad Prism, version 6.4. The statistical significance of difference between groups was analyzed using the one-way analysis of variance (ANOVA) followed by independent-sample *t* test. The level of significance was set at *P* < 0.05. The results are presented as the mean ± SEM.

## 3. Results

### 3.1. Effects of SE on Plasma Lipid Parameters

**Figure 1:**
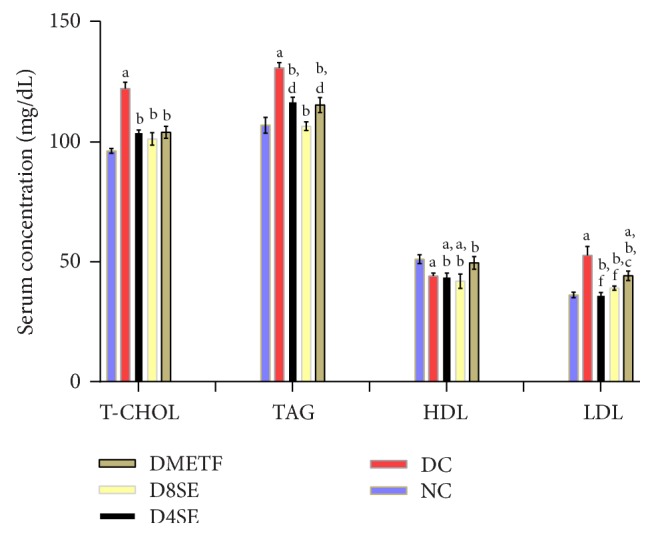
Effects of SE on lipid metabolism in STZ-treated rats. Values are expressed as mean ± SEM of 8 rats. NC: normal control, DC: diabetic control, D4SE: diabetic animals treated with SE (400 mg∖kg BW), D8SE: diabetic animals treated with SE (800 mg∖kg BW), and DMETF: diabetic animals treated with metformin (12 mg∖kg BW). a: significant when compared to NC, b: significant when compared to DC, c: significant when compared to D4SE, d: significant when compared to D8SE, and f: significant when compared to DMETF.

The effects of SE treatments on lipid homeostasis in STZ-diabetic rats are depicted by Figure 1. STZ caused notable disturbance in lipid homeostasis of rats. Relative to normal control animals. STZ control rats expressed significant increase in serum T-chol, TG, and LDL (27.31, 22.43, and 45.83%, respectively) as well as reduction in HDL (13.77%). Treatments of diabetic rats with SE extract suppressed STZ-induced hypercholesterolemia by 13.00 and 17.89% at 400 and 800 mg/kg BW, respectively. These effects were comparable to that provided by METF (15.45%). Similar pattern was noted with TAG (10.63 and 19.06%) and LDL (31.47 and 25.97%). However, SE extract failed to significantly reverse the STZ-induced decline in serum HDL.

### 3.2. Effects of SE on Adipose Tissue Triglyceride

**Figure 2:**
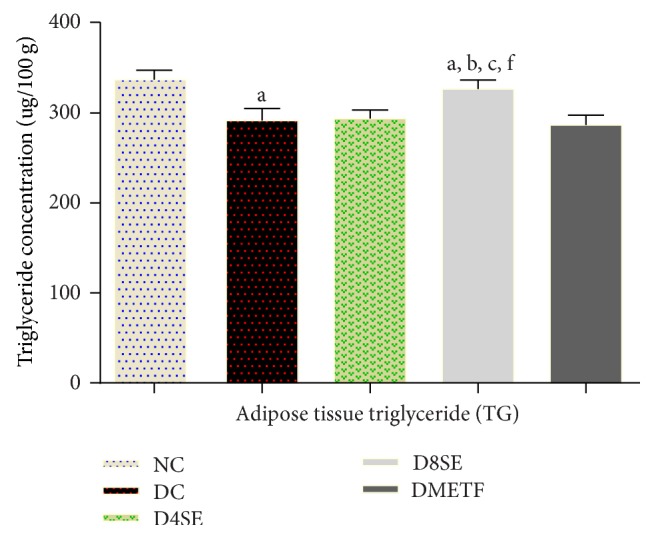
Effects of SE and METF on adipose tissue TG concentration in STZ-treated rats. Values are expressed as mean ± SEM of 8 rats. NC: normal control, DC: diabetic control, D4SE: diabetic animals treated with SE (400 mg∖kg BW), D8SE: diabetic animals treated with SE (800 mg∖kg BW), and DMETF: diabetic animals treated with metformin (12 mg∖kg BW). a: significant when compared to NC, b: significant when compared to DC, c: significant when compared D4SE d: significant when compared to D8SE, and f: significant when compared to DMETF.

Figure 2 depicts that STZ challenge in rats caused decrease in adipose tissue triglycerides (TG) by 13.38% relative to normal control animals. This change was only corrected (to near normal) in diabetic rats treated with 800 mg dosage of SE extract (pegging TG reduction to a negligible value of 1.44%). The extract at both doses compared to METF exerted significant effects on adipose tissue triglyceride content.

### 3.3. Estimation of Cardiovascular Risk Level

The effects of SE extract on cardiovascular risk predictors in terms of atherogenic index of plasma (AIP) and LDL/HDL ratio are shown in Tables 1 and 2, respectively. SE lowered AIP and LDL/HDL ratio by 57.85 and 44.12%, respectively, when the values of these factors are compared in STZ control rats and SE-treated diabetic rats, particularly at 400 mg/kg BW.

### 3.4. Phytochemicals Analysis of SE Ethanol Leaf Extract

The classes of phytochemicals present in SE ethanol leaf extract are shown in Table 3.

## 4. Discussion

One of the common complications of diabetes mellitus is dyslipidaemia [22] and the first line index of diabetic dyslipidemia is an elevated plasma level of T-chol, TG, LDL-chol, and VLDL-chol [1, 2] as well as decreased level of HDL-chol which is probably due to a fall in high density lipoprotein 2 (HDL2) [1, 23, 24]. In diabetes mellitus, due to insulin deficiency or defectiveness, glucose utilization by body cells is compromised. Though blood glucose concentration is high yet the molecules are not available to cells for energy generation. In response to the urgent energy need, fatty acids mobilization from adipose tissues is greatly enhanced, causing an obvious decrease in tissue triglyceride as observed in diabetic control rats in the current study. The attendant effect is massive flux of free fatty acids into the liver, leading to accumulation of excess fatty acids in the hepatocytes. The fatty acids are then converted to triglycerides and cholesterol and released into circulation, resulting in increased levels of plasma triglycerides and cholesterol. Secondary to the impaired ability of insulin to prevent free fatty acid release, hepatic VLDL-chol production is also increased. The Increased VLDL-chol and triglycerides cause a concomitant decrease in the level of HDL-chol and increase in the concentration of LDL-chol through activation of lipoprotein lipase and lecithin acyl-cholesterol transferase [3, 4].

In accordance, findings from the present study show that STZ caused notable disturbance in lipid homeostasis of rats. Relatively to normal control animals, diabetic control rats expressed significant elevation in serum T-chol, TG, and LDL-chol as well as reduction in HDL-chol. Nonetheless, SE moderately attenuated the upsurge in T-chol, TG, and LDL-chol. In addition, the extract at 800 mg dosage also restored the decrease in adipose tissue TG to near normal in rats, indicating that SE extract could to an extent modulate blood lipid abnormalities and maintain lipid homeostasis. The extract however failed to elicit any meaningful improvement on HDL-chol. This result supports previous reports which noted the antihyperlipidemic activity of plant materials in hyperglycaemic rats [25, 26]. The reference drug (metformin) though significantly reversed (to near normal) the HDL decline caused by STZ but its reductive effects on other lipid molecules and its ability to restore adipose tissue TG level were not significantly different from those of SE extract.

Generally, plants reported to exhibit lipid lowering activity are rich in flavonoids, alkaloids, and tannins which play significant role in the mobilization and metabolism of lipids [27, 28]. This postulation is supported by the result of the phytochemical analysis of SE leaf extract which reveals the presence of the above compounds in the extract.

Besides, atherogenic index value either in form of LDL-HDL ratio or atherogenic index of plasma (AIP) is used indirectly as an indicator of the hypolipidemic potential of a drug material or directly as predictor of cardiovascular disease (CVD) risk [29–31]. According to Dobiasova [32], AIP value range of −0.3 to 0.1 suggests low cardiovascular (CVD) risk while values between 0.1 and 0.24 indicate mild CVD risk, and values above 0.24 represent high CVD risk. In the same vein, Panagiotakos et al. [33] established that plasma LDL-HDL ratio greater than 1 is indicative of CVD risk while value lesser than 1 suggests lack of CVD risk. However, AIP which is described by the logarithm to base ten of the ratio of total triglycerides and high density lipoprotein cholesterol is often regarded as a more definite predictor.

In the present study, though the atherogenic index of plasma values, 0.28 and 0.30, shown by diabetic animals treated with SE extract are slightly above the boundary for high risk of CVD (0.24), they are nonetheless significantly lower than the 0.48 recorded for diabetic control animals and comparable to the 0.26 demonstrated by diabetic animals treated with the reference drug, metformin. Moreover, the LDL/HDL ratios in SE-treated groups were less than 1.0 (0.91 and 0.93) compared to a value of 1.28 observed in the diabetic control animals. This observation attest to the fact that SE extract in the present study showed signs of a possible role in lipid homeostasis.

More so, comparing the effect of the extract with that of metformin apparently suggests that SE extract possesses considerable antilipidaemic property. However, its effects on atherogenic markers show values which do not deviate significantly from the limits for CVD risks. It will therefore be more appropriate to describe SE leaf extract as a moderate antilipidaemic agent.

## Figures and Tables

**Table 1:** Atherogenic index of plasma (AIP).

Treatment	AIP value
Normal control	0.12 ± 0.004
STZ control	0.48 ± 0.030
SE (400 mg/kg BW)	0.28 ± 0.021
SE (800 mg/kg BW)	0.30 ± 0.070
Metf (12 mg/kg BW)	0.26 ± 0.012

Values are mean ± standard error of mean (SEM), *n* = 8. METF: metformin.

**Table 2:** LDL-HDL ratio.

Treatment	LDL/HDL
Normal control	0.61 ± 0.13
STZ control	1.28 ± 0.02
SE (400 mg/kg BW)	0.91 ± 0.21
SE (800 mg/kg BW)	0.93 ± 0.18
Metf (12 mg/kg BW)	0.83 ± 0.16

Values are mean ± standard error of mean (SEM), *n* = 8. MTF: metformin.

**Table 3:** Phytochemicals in SE extract.

Phytochemical	Result
Flavonoids	+
Alkaloids	+
Tannins	+
Saponins	−
Antraquin	+
Glycosides	+
Steroids	+
Phlobat	−
Phenols	+
Terpenoids	+

+: Present, −: absent, phlobat: phlobatannins, and antraquin: anthraquinones.
